# Light-tunable Fano resonance in metal-dielectric multilayer structures

**DOI:** 10.1038/srep33144

**Published:** 2016-09-14

**Authors:** S. Hayashi, D. V. Nesterenko, A. Rahmouni, H. Ishitobi, Y. Inouye, S. Kawata, Z. Sekkat

**Affiliations:** 1 Optics and Photonics Center, Moroccan Foundation for Advanced Science, Innovation and Research (MAScIR), Rabat 10100, Morocco; 2 Graduate School of Engineering, Kobe University, Kobe 657-8501, Japan; 3 Graduate School of Frontier Biosciences, Osaka University, Suita 565-0871, Japan; 4 Graduate School of Engineering, Osaka University, Suita 565-0871, Japan; 5 Faculty of Sciences, University Mohamed V, Rabat 10010, Morocco

## Abstract

High-Q optical Fano resonances realized in a variety of plasmonic nanostructures and metamaterials are very much promising for the development of new potent photonic devices, such as optical sensors and switches. One of the key issues in the development is to establish ways to effectively modulate the Fano resonance by external perturbations. Dynamic tuning of the Fano resonance applying the mechanical stress and electric fields has already been demonstrated. Here, we demonstrate another way of tuning, i.e., photo-tuning of the Fano resonance. We use a simple metal-dielectric multilayer structure that exhibits a sharp Fano resonance originating from coupling between a surface plasmon polariton mode and a planar waveguide mode. Using a dielectric waveguide doped with azo dye molecules that undergo photoisomerization, we succeeded in shifting the Fano resonance thorough photo-modulation of the propagation constant of the waveguide mode. The present work demonstrates the feasibility of photo-tuning of the Fano resonance and opens a new avenue towards potential applications of the Fano resonance.

It was in 1935 that Ugo Fano published a paper^1^ in which he gave a quantum mechanical interpretation of asymmetric line shapes observed in light absorption spectra of Argon, Krypton and Xenon atoms^2^. He considered the interaction between a discrete quantum level and a continuum state and succeeded in obtaining a formula that describes the asymmetric line shape with a few key parameters^1^,^3^,^4^. The asymmetric line shape, called Fano line shape, appears not only in atomic systems, but also in a variety of physical systems^4^,^5^. It is known that the Fano line shape can be well described by a classical mechanical model of coupled harmonic oscillators^6^,^7^. In recent years, Fano line shapes appearing in optical spectra of plasmonic nanostructures and metamaterials have attracted great interest and have been the subject of extensive experimental and theoretical studies^5^,^8^,^9^,^10^. Metallic nanowire arrays^11^, clusters of nanoparticles^12^,^13^,^14^,^15^,^16^, disk/ring nanocavities^17^,^18^,^19^ and metal-insulator-metal waveguides coupled to resonators^20^, are typical examples of nanostructures that exhibit the Fano resonance. The Fano resonances appearing in these nanostructures have commonly been explained in terms of interaction between a dark electromagnetic (EM) mode characterized by a sharp resonance and a bright EM mode characterized by a broad resonance.

Since the Fano-resonant nanostructures offer the opportunities for achieving high-Q resonances that induce highly enhanced electromagnetic fields in the vicinities of the nanostructures, they have potentials for achieving high performances in photonic devices, such as optical sensors and switches^5^,^8^,^10^. One of the key issues in developing such devices is the realization of dynamic tuning of the Fano resonance^10^. Cui *et al*.^21^ demonstrated mechanical tuning of Fano resonances supported by a gold heptamer structure embedded in a flexible membrane. Integrating single-layer graphene with plasmonic Fano-resonant metasurfaces, Shevets *et al*.^22^,^23^ succeeded in modulating mid-IR Fano resonances using electrostatic gating. Electric-field modulation of the Fano resonance was also demonstrated for gold nanowire gratings^24^ and silicon nanohole arrays^25^ integrated with liquid crystals. In spite of great efforts made so far, the fabrication of the nanostructures is not always easy and time consuming, preventing their real applications. Therefore, it is highly demanded to exploit structures exhibiting the Fano line shapes that can be fabricated by a low-cost, fast and easy method. Realization of the dynamic tuning of the Fano resonance in simple structures remains a challenge. In this paper, we demonstrate an alternative way of Fano resonance tuning, i.e., photo-tuning, in simple planar multilayer structures.

Longhi^26^ analyzed theoretically the Fano resonance tuning in coupled-resonator optical waveguide systems. He showed that the dynamical tuning of the Fano resonance in such systems can be achieved by modulation of the resonance frequency of one of the resonators caused by refractive index modulation. Fan *et al*.^27^ have demonstrated experimentally and theoretically that a single semiconductor nanostructure, namely a Si nanostripe, exhibits a Fano line shape in the light scattering spectrum that shifts depending on the incidence angle of light. Similar shifts of the Fano line shape in transmission spectra depending on the angle of incidence have been observed by Duempelmann *et al*.^28^ for arrays of tilted aluminum nanowires. Although these studies constitute a first step toward photo-tuning of the Fano resonance, clear experimental evidence of the photo-tuning of the Fano resonance has not been reported to date.

Very recently, we demonstrated both theoretically and experimentally the feasibility of realizing sharp Fano line shapes in attenuated total reflection (ATR) spectra of planar multilayer structures consisting of a metallic layer and dielectric layers^29^,^30^,^31^,^32^. The structures studied are simple and do not require the use of nanofabrication techniques. The physical origin of the Fano line shape in the structures has clearly been identified as the coupling between a surface plasmon polariton (SPP) mode localized at a metal-dielectric interface and a planar waveguide (PWG) mode supported by a stack of dielectric layers. In the experimental studies^31^, a poly(methyl methacrylate) (PMMA) waveguide was used to launch a PWG mode. The purpose of the present work is to extend our previous work to realize Fano resonances that can be tuned by light irradiation. To realize light-tunable Fano resonances, we use the PMMA waveguide doped with disperse red 1 (DR1) molecules instead of the pure PMMA layer.

The DR1 molecule is one of the azobenzene derivatives exhibiting *trans*-*cis* photoisomerization, and its *trans* form is known to be stable^33^,^34^,^35^,^36^,^37^,^38^. Both *trans*- and *cis*-DR1 molecules have absorption bands in the blue region of the spectrum, and they transform into each other by reversible photoisomerization as schematically shown in Fig. 1a. *Cis*-DR1 molecules revert to *trans*-DR1 molecules by thermal relaxation as well. Optical phenomena related with the *trans*-*cis* photoisomerization of azobenzene derivatives have been investigated extensively over two decades^33^,^38^, in particular for DR1 molecules embedded in PMMA matrices^34^,^35^,^36^,^37^,^39^,^40^,^41^,^42^. Linear and nonlinear optical properties of the DR1-doped PMMA films are governed by the orientation of the *trans* molecules, because they have large transition dipole moments along the long molecular axis. The optical pumping of DR1-doped PMMA films induces dramatic changes in their optical properties due to the photoisomerization, followed by thermal relaxation and random reorientation of the molecules. We demonstrated that photoinduced changes in the refractive index of the DR1-doped PMMA film can be well described by a simple model of angular hole burning (AHB)^34^, which predicts the depletion of the *trans* molecules in the direction of the polarization of pump light.

Our strategy for realizing the light-tunable Fano resonance is as follows. Since the Fano resonance in our multilayer structures arises from the coupling between the SPP and PWG modes, a change in the propagation constant of the PWG mode is thought to directly induce a shift of the Fano resonance. When the DR1-doped PMMA layer is used as a waveguide and pumped by blue light, the change in the refractive index in the waveguide may change the propagation constant of the PWG mode, thus generating a photosensitive shift of the Fano resonance. In the present paper, we demonstrate that the above strategy indeed works very well. We give clear evidence of photoinduced shifts of the Fano resonance. From a comparison of the experimental results with EM calculations, we suggest that the smallest shift of the Fano resonance observed corresponds to the change in the refractive index of the DR1-doped PMMA film smaller than 1.0 × 10^−4^.

## Results

### Photoinduced changes in ATR spectra

**Figure 1:**
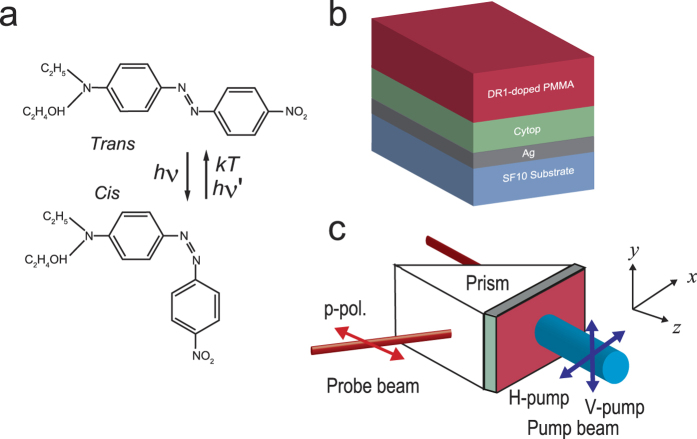
Sample and pump-probe ATR experiment. (**a**) Photoisomerization and thermal relaxation of DR1 molecule. (**b**) Multilayer sample consisting of a Ag layer, a fluorofore Cytop layer, and a DR1-doped PMMA layer, deposited on a SF10 substrate. (**c**) Kretschmann configuration of ATR measurements with pump (blue) and probe (red) beams.

The multilayer sample used in this study is schematically shown in Fig. 1b. Instead of using the pure PMMA waveguide layer as in our previous work^31^, we use the PMMA waveguide layer doped with photofunctional DR1 molecules. The sample consists of a SF10 glass substrate, a Ag layer, a fluoropolymer Cytop layer and a DR1-doped PMMA layer. The estimated thicknesses of the Ag, Cytop and DR1-doped PMMA layers are 45.5, 524 and 720 nm, respectively. As explained in detail later, the thicknesses and the dielectric constants of the layers were estimated from a theoretical fit of an experimental ATR spectrum.

To measure the angle-scan ATR spectra in a Kretschmann configuration, the multilayer sample was pasted onto the bottom surface of a 60°-prism made of SF11 glass (Fig. 1c). The prism with the sample was mounted on a computer-controlled rotating stage. P-polarized light beam from a He-Ne laser with a wavelength of 632.8 nm was used as the probe beam. The diameter of the probe beam is ~2 mm. The ATR spectra were measured as a function of the angle of incidence, *θ*. The precision of the angle of incidence (internal angle inside the prism) in the present measurements is around 0.018°. To perform pumping experiments on the present sample, a pump beam with a wavelength of 488.0 nm from a semiconductor diode laser was directed onto the sample surface as schematically shown in Fig. 1c. To assure the overlap between the pump beam and the probe beam, the pump beam as large as ~7 mm in diameter was used. The polarization of the pump beam was set to either the vertical or horizontal direction (V-pump or H-pump).

**Figure 2:**
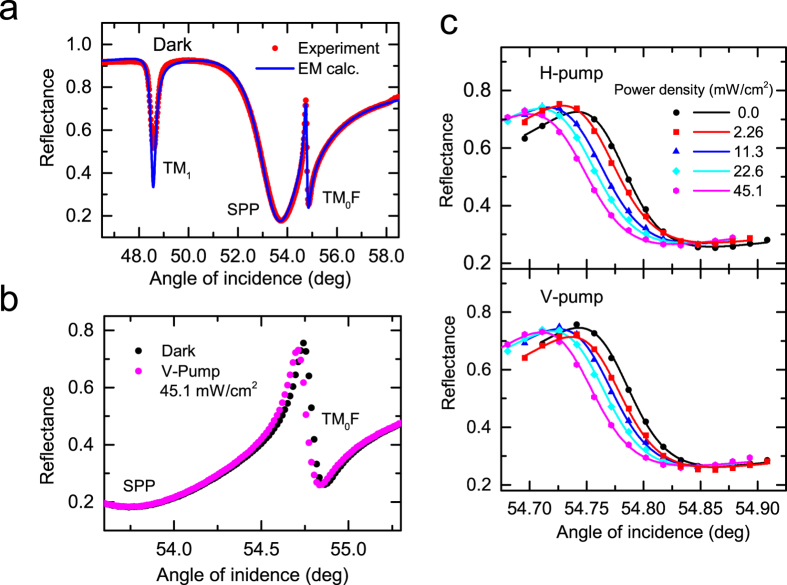
Results of pump-probe ATR measurements. (**a**) Experimental (dots) and theoretical (solid curve) ATR spectra of a sample without pump irradiation. (**b**) Experimental ATR spectra in a narrower angle region measured without and with pump irradiation. The Fano resonance shifts to a lower angle under irradiation, while SPP resonance stays at the same position. (**c**) Results of systematic pumping experiments performed with various pumping power under H- and V-pump conditions. The pump power densities are estimated from the measured power and diameter of the pump laser beam.

In Fig. 2a, we show a typical *θ*-scan ATR spectrum observed for a sample with a DR1-doped PMMA waveguide without pump irradiation (under dark condition). The spectrum is very similar to that obtained for the sample without DR1 doping reported in our previous paper^31^. We see clearly the Fano line shape appearing around 54.7° (denoted as TM_0_F in the figure), which is due to the coupling between the SPP mode at the Ag/Cytop interface and the TM_0_ PWG mode supported by the DR1-doped PMMA waveguide. We see also a dip around 48.5° (denoted as TM_1_) corresponding to the excitation of the TM_1_ PWG mode. The dip appearing around 53.7° (denoted as SPP) corresponds to the excitation of SPP mode at the Ag/Cytop interface. In the figure, a theoretical ATR spectrum obtained by the EM calculation is also presented. In the calculation, a value of the dielectric constant of SF11 at *λ* = 632.8 nm, *ε*_p_ = 3.1634, was taken from a data base^43^. We searched for values of the thickness and dielectric constant of the Ag, Cytop and PMMA layers that reproduce well the experimental spectrum. The solid curve in Fig. 2a was generated by a set of parameters: *s* = 45.5 nm and *ε*_Ag_ = −16.5837 + *i*2.3417 for the Ag layer, *t* = 524 nm and *ε*_Cytop_ = 1.8252 + *i*8.1100 × 10^−3^ for the Cytop layer, and *d* = 720 nm and *ε*_PMMA_ = 2.2320 + *i*2.9880 × 10^−4^ for the PMMA layer. We see that the experimental spectrum is well reproduced by the EM calculation.

A remarkable feature observed in the present sample is the shift of the Fano resonance caused by light irradiation. In Fig. 2b, we compare, in an expanded angle scale, the dark spectrum with a spectrum obtained under V-pump irradiation with a power density of 45.1 mw/cm^2^. In the pumping experiments, the pump beam was aligned to be approximately normal to the sample surface, when the angle of incidence of the probe beam scans the narrow region of the Fano resonance between 54.70 to 54.80°. We see clearly that in the presence of the pump beam, the Fano line shape is shifted to a lower angle, while the SPP dip remains at the same angle. Results of a systematic pumping experiment performed under the V- and H-pump conditions are presented by dots in Fig. 2c. The power densities of the pump beam calculated from measured powers and the beam diameter (~7 mm) are given in the figure. Figure 2c demonstrates that the Fano resonance shifts more and more to lower angles as the power density increases under both the V- and H-pump conditions. We note here that the shift of the Fano resonance was not observed for samples not containing the DR1 molecules. Therefore, the shift observed in the present sample is caused by the DR1 molecules doped into the PMMA waveguide.

**Figure 3:**
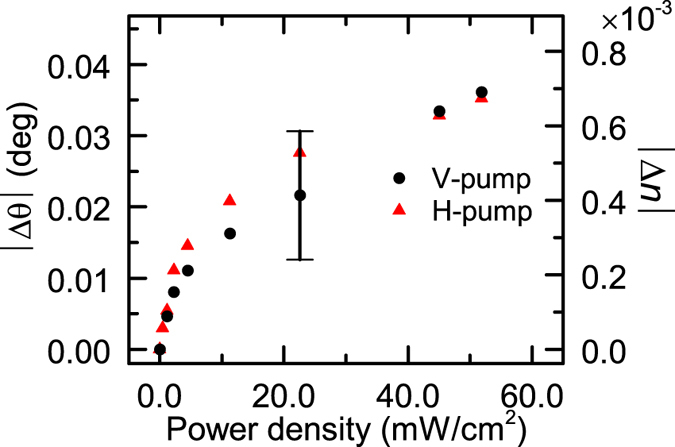
Photoinduced shift of the Fano resonance angle. The left vertical axis represents the shift of the Fano resonance angle. The angles were determined from the fits of experimental line shapes to the Fano line shape function. The vertical bar indicates the angular precision of the present measurements. The observed shift was reproduced by EM calculations of the ATR spectra assuming the change in the refractive index of the DR1-doped PMMA layer. The right vertical axis is scaled by the change in the refractive index that reproduces the observed shift.

To analyze the observed shift in a systematic way, we fitted the experimental line shapes to a Fano line shape function modified from its initial form^3^,^5^. Since the initial form is given as a function of energy or frequency, we modify it to obtain the line shape as a function of the angle of incidence *θ* as,
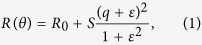
 where *q* is the so-called Fano factor, which describes the degree of the asymmetry in the line shape; *ε* is defined as 2(*θ* − *θ*_0_)/*γ* by the resonance angle *θ*_0_ and the width *γ*. *R*_0_ is introduced to take into account the nonzero minimum value of the reflectance and *S* is a scale factor. The solid curves in Fig. 2c are the fits to the experimental data. The fits were obtained with negative values of *q* falling in a range between −1.558 and −1.690. From the resonance angles determined from the Fano fits, we define the shift of the resonance as Δ*θ* = *θ*_*irrad*_ − *θ*_*dark*_, where *θ*_*irrad*_ and *θ*_*dark*_ are the resonance angles obtained with and without pump irradiation. In Fig. 3, the absolute value of the shift |Δ*θ*| is plotted as a function of the pump power density for both the V- and H-pump conditions. The vertical bar in the figure indicates the precision of the present angular measurements (for the internal angle inside the prism). The figure demonstrates that the shift increases rather rapidly in the low power density region and tends to saturate in the high power density region. There is no discernible difference between the V- and H-pump results that exceeds the present angular precision.

The shift of the Fano resonance presently observed under pump irradiation is due to the photoisomerization of the DR1 molecules. When a *trans* molecule is subject to the optical pumping, the probability of the *trans*-*cis* photoisomerization is proportional to cos^2^*θ*_p_, where *θ*_p_ is the angle between the molecular transition dipole moment and the electric field of the pump light (direction of polarization)^34^,^36^,^37^,^39^. It turns out that the *trans* molecules oriented closer to the direction of pump polarization have higher probabilities of the *trans*-*cis* transformation. Before pump irradiation, the DR1 molecules are randomly oriented in the PMMA layer. Under polarized light irradiation, the polarization sensitive *trans*-*cis* photoisomerization leads to a selective depletion of *trans* molecules along the direction of pump polarization, i.e., AHB. The depletion of the *trans* molecules results in the decrease in the refractive index of the sample along the direction of pump polarization (anisotropic change in the refractive index)^34^,^37^,^39^. The decrease in the refractive index of the DR1-doped PMMA waveguide may decrease the in-plane wavenumber of the PWG mode finally resulting in the shift of the Fano resonance to a lower angle.

### Real-time measurements of photoinduced changes

**Figure 4:**
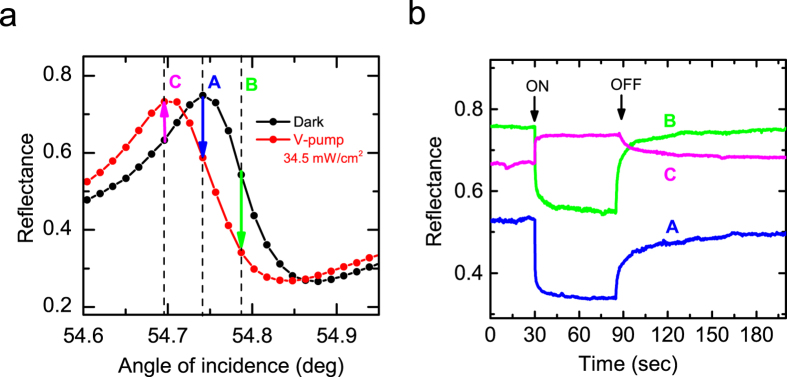
Results of real-time on-off experiments. (**a**) Steady-state Fano line shapes observed without and with pump irradiation. Vertical broken lines A, B and C indicate the angles of incidence for the probe beam fixed in the real-time on-off measurements. (**b**) Time evolutions of reflectance signals obtained for the angles A, B and C.

To check whether the presently observed photoinduced shift of the Fano resonance is due to the photoisomerization of DR1 molecules, we performed real-time measurements of ATR signals by switching on and off the pump beam. A V-pump beam with a power density of 34.5 mW/cm^2^ was incident on the sample with an angle of incidence of ~45°. In Fig. 4a we show the steady-state Fano line shapes observed without and with the pump beam. To monitor the reflectance with fixed angles of incidence for the probe beam, we selected three different angles indicated by the vertical broken lines, which correspond to the maximum (A), high-angle side (B) and low-angle side (C) of the maximum in the Fano line shape obtained under the dark condition, respectively. Figure 4b shows time evolutions of the reflectance obtained for the three different angles. When the pump beam is switched on, the reflectance signal decreases for the angles A and B, while the signal increases for the angle C. The behaviors of the signals are in good agreement with those predicted from the photoinduced shift of the Fano resonance as indicated by arrows in Fig. 4a. Upon switching on the pump, the signals change rather fast with a time constant less than 1.0 sec. When the pump is switched off, the signals tend to relax to the initial dark values. This process is relatively slow with a time constant of several 10 seconds. It should be noted that the transients of the reflectance signals seen in Fig. 4b are very similar to those of absorbance and ATR signals reported in previous studies on the photoisomerization in DR1-doped PMMA films^34^,^36^,^37^,^39^. The close similarity allows us to attribute the present photoinduced shift of the Fano resonance to the photoisomerization of the DR1 molecules dispersed in the PMMA layer. As described in detail in our previous papers^35^,^36^,^39^,^40^, thermal effects can be ruled out as the origin of the observed shift.

## Discussion

To discuss more in detail the present photoinduced changes in the Fano resonance, we introduce a Cartesian coordinate attached to the sample-prism system as shown in Fig. 1c. In this coordinate system, the plane of incidence for the pump and prob beams lies in the *x*–*z* plane. The polarization of the probe beam was set to the p-polarization; corresponding electric fields have thus *x* and *z* components, *E*_*x*_ and *E*_*z*_, respectively. When the probe beam scans the narrow region of the Fano resonance, the pump beam is almost normal to the sample surface. Therefore, the electric field of the pump light can be assumed to have only the *y* component (*E*_*y*_) for V-pump, and only the *x* component (*E*_*x*_) for H-pump. Since the AHB induces the optical anisotropy in the DR1-doped PMMA layer, we introduce anisotropic refractive indices of the layer *n*_*x*_, *n*_*y*_ and *n*_*z*_. The ATR spectra are determined only by *n*_*x*_ and *n*_*z*_, because the probe light is p-polarized. According to a simple model of AHB^34^,^39^, the photoisomerization induces anisotropic changes in the refractive indices described by Δ*n*_‖_ and Δ*n*_⊥_, where Δ*n*_‖_ and Δ*n*_⊥_ represent the changes in the directions parallel and perpendicular to the polarization of the pump light, respectively. The AHB model predicts that low irradiation intensities produce a change of refractive index Δ*n*_‖_ = 3Δ*n*_⊥_, while for high irradiation intensities, saturation prevails and the ratio Δ*n*_‖_/Δ*n*_⊥_ tends towards 1. A detailed theoretical description of the AHB model can be found in our previous papers^34^,^37^.

**Figure 5:**
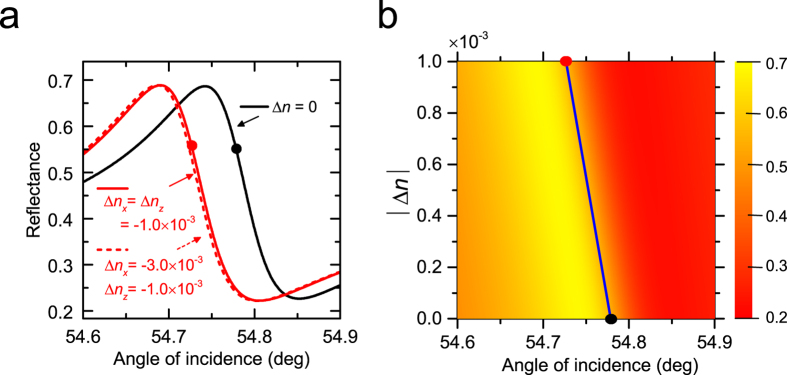
Shift of Fano resonance reproduced by EM calculations. (**a**) Fano line shapes theoretically obtained to reproduce the experimental shift. Filled circles represent the resonance angles determined from the Fano fits. (**b**) Contour plot of the Fano line shape obtained by varying continuously the refractive index of the DR1-doped PMMA layer. The solid line gives the dependence of the resonance angle on the change in the refractive index. The result presented by the solid line allows us to convert the observed shift of the resonance angle into the change in the refractive index.

We performed EM calculations to examine the influence of the change in the refractive index of the DR1-doped PMMA waveguide on the ATR Fano line shape. The fitting parameters for the dark spectrum (Fig. 2a) were used as initial parameters. In case of V-pump, the pump *E*_*y*_ fields may induce the changes in *n*_*x*_ and *n*_*z*_ given by Δ*n*_*x*_ = Δ*n*_*z*_ = Δ*n*_⊥_. We simply write as Δ*n* = Δ*n*_*x*_ = Δ*n*_*z*_ = Δ*n*_⊥_. ATR spectra in the region of the Fano resonance obtained for Δ*n* = 0 and −1.0 × 10^−3^ are presented in Fig. 5a as solid lines. We see that the spectrum calculated with Δ*n* = −1.0 × 10^−3^ exhibits a low-angle shift similar to that observed experimentally under optical pumping. For H-pump, the pump *E*_*x*_ fields may induce the changes given by Δ*n*_*x*_ = Δ*n*_‖_ and Δ*n*_*z*_ = Δ*n*_⊥_, respectively. In the limit of high pump intensity, we recover the V-pump case, since Δ*n*_‖_ = Δ*n*_⊥_ holds as mentioned above. In the limit of low pump intensity, since Δ*n*_‖_ = 3Δ*n*_⊥_ holds, we have Δ*n*_*x*_ = Δ*n*_‖_ = 3Δ*n*_⊥_ = 3Δ*n* and Δ*n*_*z*_ = Δ*n*_⊥_ = Δ*n*. The broken curve in Fig. 5a is the Fano line shape obtained with this assumption for Δ*n* = −1.0 × 10^−3^. We see that the shift of the Fano line shape relative to that of the high pump intensity limit or V-pump case is very small and well below the angular precision of the present measurements. This is in good agreement with the experimental results presented in Fig. 3, where no appreciable difference between the V-pump and H-pump results exceeding the present angular precision is observed. These facts allow us to simplify our theoretical analysis; in what follows, we present calculated results only for the case of Δ*n*_*x*_ = Δ*n*_*z*_ = Δ*n*.

Figure 5b shows a contour plot of the Fano line shape obtained by continuously varying Δ*n* from 0 to −1.0 × 10^−3^. The calculated Fano line shapes were fitted to the Fano formula (equation (1)) to determine the Fano resonance angles. The solid line in Fig. 5b represents the shift of the Fano resonance angle as a function of |Δ*n*|. We can define an angular sensitivity of the Fano line shape to the variation of the refractive index as 
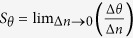
, where Δ*θ* is the shift of the resonance angle caused by the refractive index change Δ*n*. From the slope of the solid line in Fig. 5b we obtain a value *S*_*θ*_ = −52.27 deg RIU^−1^. Using this value of *S*_*θ*_, we can convert the observed shift |Δ*θ*| plotted in Fig. 3 to the change in the refractive index |Δ*n*|. The right vertical axis of Fig. 3 was scaled with |Δ*n*| converted form |Δ*θ*|. The figure demonstrates that the optical pumping of the present sample with a pump power density of up to ~50 mW/cm^2^ induces the change in the refractive index of up to ~0.7 × 10^−3^. The amount of change in the refractive index is in good agreement with experimental results reported so far for similar DR1-doped PMMA films^34^,^36^,^37^,^39^. The good agreement further confirms that the present shift is caused by the photoisomerization of DR1 molecules.

In conclusion, we have demonstrated the feasibility of realizing light-tunable Fano resonances in planar metal-dielectric multilayer structures. The photosensitivity was brought by photofunctional DR1 molecules embedded in PMMA waveguides. Upon blue light irradiation, the DR1 molecules undergo the *trans*-*cis* photoisomerization resulting in the change in the refractive index of the DR1-doped PMMA waveguide. The change in the refractive index induces the change in the propagation constant of the PWG mode, finally leading to the photoinduced shift of the Fano resonance in the ATR spectra. Analyses of the shift based on the EM calculations allowed us to estimate the photoinduced change in the refractive index; the observed shift corresponds to the change in the refractive index that falls in a range of 1.0 × 10^−4^. The results of real-time on-off measurements of the reflectance presented in Fig. 4c suggest straightforward applications of the photo-sensitive Fano resonance to optical switches and modulators. The fine tuning of the Fano resonance achieved by controlling the pump light intensity may allow the observation of an extremely narrow Fano resonance predicted by our previous simulations^32^, which is not possible by a mechanical angle scan using a stepping motor. Operations in such an optical scan mode may open a new avenue for realizing optical sensors with extremely high sensitivities as well as other active optical elements with high performances.

## Methods

### Sample preparation

To prepare the multilayer samples (Fig. 1c), first a Ag film of approximately 45 nm in thickness was deposited on a cleaned SF10 glass substrate by a vacuum evaporation technique. The deposition rate was 0.1 nm/sec. A fluoropolymer Cytop film was then spin coated on the Ag film; Cytop solution of ~6 wt% was used with a rotation speed of 3,000 rpm. To remove the remaining solvent, the sample was baked in air at 140 °C for 30 min. To complete the sample a poly(methyl methacrylate) (PMMA) film doped with DR1 molecules was spin coated on top of the Cytop film; toluene solution of a mixture of PMMA and DR1 with a concentration of ~6 wt% was spun with a rotation speed of 5,000 rpm. The sample was again baked in air at 140 °C for 30 min. The concentration of DR1 relative to PMMA was ~5 wt%.

### Pump-probe ATR measurements

The angle-scan ATR spectra were measured by pasting the multilayer sample onto the bottom surface of a 60°-prism made of SF11 glass with the aid of index matching oil (Fig. 1d) (Kretschmann configuration). The prism with the sample was mounted on a computer-controlled rotating stage. Light from a He-Ne laser with a wavelength of 632.8 nm (probe beam) was incident on the prism through a chopper. The diameter of the probe beam is ~2 mm. The intensity of the reflected light was measured as a function of the angle of incidence, using a Si photo-diode connected to a lock-in-amplifier. The reflectance spectra were obtained by normalizing the intensity data recorded with the sample to that recorded for a bare part of the prism. The precision of the incident angle (internal angle inside the prism) in the present measurements is around 0.018°. To perform optical pumping, a pump beam with a wavelength of 488.0 nm from a semiconductor diode laser was directed onto the sample surface (Fig. 1c). The light beam from the laser was first expanded by a beam expander to generate a beam ~10 mm in diameter and the central part of the beam after passing through an attenuator and a polarizer was selected by an aperture to generate a final pump beam ~7 mm in diameter. The power of the pump beam after the aperture was measured by a power meter; the power was varied from 0 to 25 mW. The polarization of the pump beam was set to either the vertical or horizontal direction (V-pump or H-pump). The angle of incidence for the pump beam was adjusted to be normal to the sample surface, when the angle of incidence for the probe beam scans a narrow angle region of the Fano resonance.

We performed a systematic measurement of the dependence of the Fano resonance on the pump power in the following manner. First, ATR spectra were measured without pump irradiation. Then, the pump laser power was adjusted to a minimum value in a series of the measurements and the sample was exposed to the pump beam. After waiting at least ~5 min to achieve the steady state for the adjusted laser power, ATR scans were started. When the ATR measurements for the initial pump power were completed, we raised the pump power to a next desired value, kept the sample under pump irradiation for at least ~5 min and then restarted the ATR measurements. We repeated the procedures until the pump power reaches a maximum value.

We also performed transient measurements of the Fano resonance switching on and off the pump irradiation by a shutter. Before starting the transient measurements, steady-state ATR spectra were measured without and with pump irradiation. Based on the results, the angles of incidence for the probe beam to be fixed in the transient measurements were selected. After setting the angle of incidence at one of the selected angles, the sample was kept under dark condition at least for 2 hours to erase the effects of previous pump irradiation. Then the real-time measurement of the reflectance signal is started and at a certain moment the pump beam was switched on. After about 60 sec, the pump beam was switched off, while continuing the real-time measurement for more than 5 min. Fixing the angle of the incidence at another one, we repeated the same procedure.

### Electromagnetic calculation of ATR spectra

The ATR spectra were calculated using freely available Winspall software package. The package allows fitting of the measured ATR spectrum to the calculated one to estimate the parameters (thicknesses and dielectric constants) of layers in the multilayer structure. The package is also applicable to layers having anisotropic refractive indices. To obtain the theoretical spectra, the SF10 substrate used in the experiments was omitted. When the SF10 substrate is incorporated into the calculations, small fluctuations in the reflectance appear in high angle regions of the ATR specra. The fluctuations are due to the interference of multiply reflected light inside the substrate. Since such fluctuations are not observed in experiments, presumably due to the smearing out of the interference caused by surface roughness of the substrate, theoretical spectra were calculated without the SF10 substrate to better reproduce the experimental spectra.

## Additional Information

**How to cite this article**: Hayashi, S. *et al*. Light-tunable Fano resonance in metal-dielectric multilayer structures. *Sci. Rep.* **6**, 33144; doi: 10.1038/srep33144 (2016).
